# Reconstruction of gene regulatory networks from single cell transcriptomic data

**DOI:** 10.18699/vjgb-24-104

**Published:** 2024-12

**Authors:** M.A. Rybakov, N.A. Omelyanchuk, E.V. Zemlyanskaya

**Affiliations:** Institute of Cytology and Genetics of the Siberian Branch of the Russian Academy of Sciences, Novosibirsk, Russia Novosibirsk State University, Novosibirsk, Russia; Institute of Cytology and Genetics of the Siberian Branch of the Russian Academy of Sciences, Novosibirsk, Russia; Institute of Cytology and Genetics of the Siberian Branch of the Russian Academy of Sciences, Novosibirsk, Russia Novosibirsk State University, Novosibirsk, Russia

**Keywords:** gene regulatory network, single-cell data, RNA sequencing, scRNA-seq, scATAC-seq, регуляторная генная сеть, данные для отдельных клеток, секвенирование РНК, scRNA-seq, scATAC-seq

## Abstract

Gene regulatory networks (GRNs) – interpretable graph models of gene expression regulation – are a pivotal tool for understanding and investigating the mechanisms utilized by cells during development and in response to various internal and external stimuli. Historically, the first approach for the GRN reconstruction was based on the analysis of published data (including those summarized in databases). Currently, the primary GRN inference approach is the analysis of omics (mainly transcriptomic) data; a number of mathematical methods have been adapted for that. Obtaining omics data for individual cells has made it possible to conduct large-scale molecular genetic studies with an extremely high resolution. In particular, it has become possible to reconstruct GRNs for individual cell types and for various cell states. However, technical and biological features of single-cell omics data require specific approaches for GRN inference. This review describes the approaches and programs that are used to reconstruct GRNs from single-cell RNA sequencing (scRNA-seq) data. We consider the advantages of using scRNA-seq data compared to bulk RNA-seq, as well as challenges in GRN inference. We pay specific attention to state-of-the-art methods for GRN reconstruction from single-cell transcriptomes recruiting other omics data, primarily transcription factor binding sites and open chromatin profiles (scATAC-seq), in order to increase inference accuracy. The review also considers the applicability of GRNs reconstructed from single-cell omics data to recover and characterize various biological processes. Future perspectives in this area are discussed.

## Abstract (ru)

Генные регуляторные сети – интерпретируемые графовые модели регуляции экспрессии генов – являются важным инструментом для понимания и исследования механизмов, которые клетки реализуют в процессе развития и при ответе на различные внутренние и внешние стимулы. Исторически первый подход для реконструкции генных регуляторных сетей основывался на анализе литературных сведений, в том числе обобщенных в базах данных. В настоящее время основной способ системной реконструкции генных регуляторных сетей – анализ омиксных (в первую очередь транскриптомных) данных; разработан ряд математических подходов для решения этой задачи. Развитие технологий получения омиксных данных для отдельных клеток сделало возможным проведение широкомасштабных молекулярно-генетических исследований с беспрецедентно высоким уровнем разрешения. В частности, появилась возможность реконструировать генные регуляторные сети для отдельных клеточных типов и для различных стадий развития клеток. Однако технические и биологические особенности омиксных данных отдельных клеток требуют специальных программ для решения этой задачи. В обзоре описаны подходы и программы, которые разработаны и используются для построения генных регуляторных сетей по транскриптомным данным отдельных клеток (scRNA-seq). Разбираются преимущества применения транскриптомных данных для отдельных клеток по сравнению с транскриптомами многоклеточных образцов а также их недостатки в рамках решения задачи реконструкции регуляторных генных сетей. Существенное вни- мание уделяется повышению точности генных регуляторных сетей, построенных по транскриптомным данным отдельных клеток с помощью привлечения других омиксных данных, в первую очередь данных по сайтам связывания транскрипционных факторов и профилирования районов открытого хроматина (scATAC- seq). Рассматриваются вопросы применимости получаемых сетей в молекулярно-генетических исследованиях, приводятся примеры успешного использования генных регуляторных сетей, реконструированных различными методами с применением омиксных данных отдельных клеток для решения конкретных биологических задач. Обсуждаются перспективные направления развития этой области.

## Introduction

**Fig. 1.**
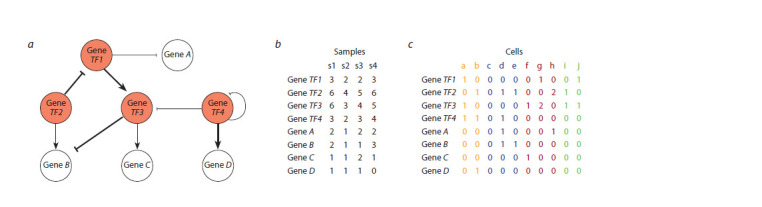
Gene regulatory network and transcriptomic data behind its construction. а – visualization of a GRN graph model; b – gene expression matrix constructed from bulk RNA-seq data for several samples (s1–s4); c – gene expression matrix constructed from scRNA-seq data for a single sample. The graph nodes denote genes, edges reflect regulatory links, including their direction, type (activation or inhibition of transcription), and magnitude (the larger the weight of the edge, the stronger the regulator’s influence on transcription). Red nodes correspond to TF-coding genes, white nodes correspond to other genes. In the GRN, edges originate only from TF-coding genes. In panel (c), different colors denote different cell types.

A gene network is a group of coordinately expressed genes that interact with each other through the RNAs and proteins they encode, as well as the products of protein activity (Kolchanov et al., 2013). Gene networks are a central object of systems biology. To explore specific aspects more deeply, specialized types of gene networks are distinguished. Among them, gene regulatory networks (GRNs) hold a special place, as they describe the regulation of gene expression by transcription factors (TFs) – a key mechanism for a flexible implementation of genetic information (Huynh-Thu, Sanguinetti, 2019). GRNs are visualized as graphs of interactions between TFs and the genes they regulate (Fig. 1a) (Badia-i-Mompel et al., 2023). Each node in a GRN represents a gene (some of which encode TFs), while edges correspond to regulatory relationships between TF-encoding genes and other genes (these relationships may reflect true molecular interactions between TFs and promoters of their target genes or merely their statistical correlation). An edge may have a sign indicating whether it describes activation or inhibition of transcription, and a weight reflecting the strength of the regulator’s influence. Thus, GRNs represent models of the logic of regulatory events between genes during execution of cellular programs (Tieri, Castiglione, 2021). They provide a viable alternative to classical modeling with differential equations when kinetic information is unavailable.

GRNs can be constructed based on information about TFs and their target genes from publications or inferred de novo from transcriptomic data (Badia-i-Mompel et al., 2023). Bulk RNA-seq results in expression levels for each gene aggregated across all cells in a tissue or organ sample. Bulk RNA-seq data can be presented as a so-called expression matrix, which provides the expression values for each gene (depicted in lines) across different samples (depicted in columns) (Fig. 1b). Given that gene expression levels in these matrices result from regulation mediated by TF binding to gene promoters, a mathematical model can be constructed to explain the observed gene expression levels (Mercatelli et al., 2020; Nguyen et al., 2021). Most GRN inference methods designed for transcriptomic data are based on this premise (Mercatelli et al., 2020). Currently, GRN reconstruction from RNA-seq data is one of the topics in systems biology, within which a large number of methods and software programs have been developed (Nguyen et al., 2019; Mercatelli et al., 2020).

At the same time, the approach described above has drawbacks. First, transcriptomic data do not contain explicit information about specific regulatory events (e. g., TF binding to the promoters of the genes they regulate); all TF-target links are mathematically inferred from gene expression levels. As a result, non-existent (erroneous) connections may be reconstructed. Incorporating data that directly describe transcriptional regulation (e. g., genome-wide open chromatin profiles or TF binding sites) can significantly improve GRN accuracy (Sönmezer et al., 2020; Isbel et al., 2022). Second, RNA-seq data do not account for the heterogeneity of cell populations, whereas gene expression can vary dramatically among different cell types. This issue is addressed by scRNA- seq (Tang et al., 2009).

Single cell transcriptomic data represent an expression matrix where lines correspond to genes and columns correspond to cells (Fig. 1c), which can be grouped by cell types using special approaches (Luecken, Theis, 2019). scRNA-seq opens up opportunities to investigate biological processes at the level of individual cell types and provides new perspectives for GRN reconstruction and analysis (Nguyen et al., 2021). GRNs for individual cell types will allow the discovery of regulatory circuits specific to cell states or degrees of differentiation.

In this review, we discuss methods for GRN inference from scRNA-seq data, with a detailed focus on the incorporation of other omics data, primarily TF binding sites and open chromatin profiles. Special attention is given to biological results that have been achieved through GRN analysis.

## Single-cell transcriptomes as a data source for GRN inference

Besides enabling inference of cell-type specific GRNs, scRNA-seq data offer other advantages over bulk RNA-seq. Since the number of interactions within a GRN is typically quite large, a substantial number of transcriptomic profiles (columns in the expression matrix, Fig. 1) is required for their accurate reconstruction. This is not always achievable with bulk RNA-seq data (Fig. 1b) (Altay, 2012), whereas scRNA-seq data contain a representative set of transcriptomes (ranging from several hundred to several thousand) (Fig. 1c) (Luecken, Theis, 2019).

The ultimate purpose of GRNs is to outline the dynamics of gene expression regulation in biological processes, including cell differentiation and responses to various internal and external stimuli. For the most accurate GRN inference from bulk RNA-seq data, time series experiments are required. In contrast, scRNA-seq data from one sample can contain information about gene expression changes over time if cells within the sample participate in the same biological process (e. g., differentiation) and are undergoing different stages (Saelens et al., 2019; Hou et al., 2023). In such cases, computational positioning of cells along a pseudotime trajectory (with the order of cells defined by the distance between their transcriptomes) allows for a good approximation of gene expression dynamics throughout the process

However, it is important to remember that, in some samples, cells may be in the same state or they can participate in numerous independent processes, making reconstruction of biologically meaningful pseudotime trajectories impossible (Pratapa et al., 2020). Therefore, when selecting a method for GRN inference, it is crucial to determine whether pseudotime information is present in the single-cell transcriptome dataset, as some methods are designed specifically for data with cellular dynamics, while others are only suitable for static data. There are also methods that can be applied to both types of data.

At the same time, scRNA-seq data have some features that complicate their analysis, in particular, GRN reconstruction (Wagner et al., 2016; Nguyen et al., 2021). These concern transient activation or low expression of certain genes, gene expression changes during cell cycle, and other factors. The widespread use of scRNA-seq technology in biology has led to development of multiple algorithms for analyzing the data it generates, each addressing these challenges in different ways.

## Reconstruction of GRNs from scRNA-seq data

**Fig. 2.**
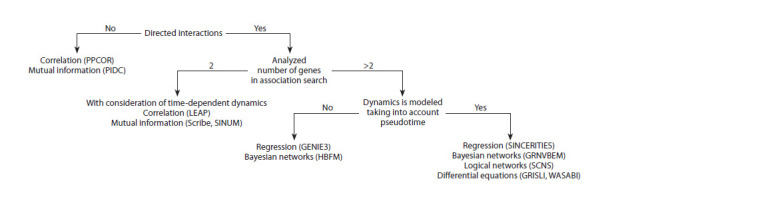
The main categories of popular algorithms used for GRN inference from scRNA-seq data.

In this section, we describe the main categories of popular algorithms used for GRN inference from scRNA-seq data (correlation- and mutual information-based methods, regression, Bayesian and logical networks, mathematical modeling with differential equations) (Fig. 2). It is worth noting that in benchmarking of GRN inference tools on both simulated and real scRNA-seq data, no single method has proven to be universally superior (Chen, Mar, 2018; Blencowe et al., 2019; Pratapa et al., 2020). Such variability may be attributed to the fact that each method is suitable for specific types and sources of data for which it was developed.

Correlation-based algorithms

Pearson correlation, a widely recognized statistical index for calculating the association between two variables, has been applied to measure the co-expression of TF-coding genes and their potential targets in RNA-seq and scRNA-seq datasets (Hong et al., 2013; Nguyen et al., 2021). Being symmetric in its arguments, correlation does not predict the directionality of regulatory interactions. It can identify associations between pairs of genes that do not necessarily have a direct regulatory relationship. Methods such as PPCOR (Kim, 2015) account for the influence of other genes by calculating semi-partial correlation coefficients. LEAP (Specht, Li, 2017), an algorithm specifically designed for the analysis of single-cell data, computes the maximum Pearson correlation between each pair of genes over varying lag-windows, given that the cells were arranged in a pseudotime order. Since this type of correlation is not symmetric, LEAP is capable of reconstructing directed gene regulatory networks. As a result of testing this program on transcriptomes from 564 individual mouse dendritic cells, LEAP identified several thousand previously unknown links between genes (Shalek et al., 2014).

Mutual information-based algorithms

Information-theoretic approaches utilize mutual information, which measures the reduction in entropy for one variable (e. g., the expression level of one gene) given the value of another variable (e. g., the expression level of another gene) (Chan et al., 2017; Qiu et al., 2020; Chang et al., 2024). To reduce false positives arising from indirect interactions between two genes, methods such as PIDC (Chan et al., 2017) use partial information decomposition (PID) to compute the proportional unique contribution (PUC) for a pair of genes that cannot be explained by the expression of a third gene. Since this relationship is symmetric, the reconstructed edges are undirected.

PIDC has been successfully applied to reconstruct GRNs from single-cell transcriptomes for three processes in mice: differentiation of megakaryocytes and erythrocytes from a common precursor, early embryogenesis, and embryonic hematopoiesis. In all three examples, PIDC identified previously unknown links, effectively highlighted gene modules at different stages of differentiation, and suggested gene interactions that facilitate transitions between stages. In a systematic evaluation of 12 different GRN inference tools, PIDC was identified as one of the most effective (Pratapa et al., 2020).

Scribe (Qiu et al., 2020) uses pseudotime to compute restricted directed information (RDI). This measure assesses the mutual information between the preceding expression level of a TF-coding gene and the current expression level of a target gene, which is conditioned by the regulator expression earlier in the pseudotime series. Since the mutual information between preceding and current expression is asymmetric, Scribe can infer directed edges. Scribe has been applied both for verifying the existence of individual connections in various gene networks and for inferring the GRN of early embryogenesis in Caenorhabditis elegans, where the known hierarchy of transcriptional regulation of genes was reproduced.

The third program, SINUM, which also evaluates mutual information between any two genes and determines whether they are dependent or independent in a specific cell, has been tested on various types of data and has shown high effectiveness in identifying cell types, their marker genes, and gene connections, as well as in studying changes in gene associations during the differentiation of human embryonic stem cells into endoderm (Chang et al., 2024).

Regression-based algorithms

GRNs can be reconstructed by modeling the expression of each gene as a function of the expression levels of other genes and solving the resulting system of equations by using regression-based methods (Huynh-Thu et al., 2010; Gao et al., 2017; Moerman et al., 2018). GENIE3 employs a random forest method, which is based on an ensemble of regression trees (Huynh-Thu et al., 2010). The weight of the edge from a TF to a target gene arises from the significance of the TF in predicting the expression of the target gene, averaged across all regression trees in the random forest. GENIE3 was developed and has been widely used for bulk RNA-seq data analysis. The GRNBoost2 software enhances the scalability of GENIE3, particularly in terms of efficiently processing large datasets from single cells (Moerman et al., 2018). Both GENIE3 and GRNBoost2 have demonstrated their effectiveness in reconstructing GRNs from single-cell transcriptomes, showing good overlap with known biological interactions (Kang et al., 2021).

The SINCERITIES algorithm was specifically designed for single-cell transcriptomes and solves a regression model, which is based on temporal or pseudo-temporal changes in the distributions of gene expression levels (Gao et al., 2017). GRNBoost2 and SINCERITIES have been identified among the most effective algorithms for GRN inference in benchmarking of 12 programs based on different types of modeling (Pratapa et al., 2020). However, a recent comparative analysis of performance across different datasets and metrics revealed that GRNBoost2 generally outperforms SINCERITIES and more accurately identifies hubs in GRNs (Stock et al., 2024).

Bayesian networks

Another GRN inference approach models regulatory interactions within a Bayesian network. The GRNVBEM algorithm works with time samples, i.e. it requires that cells be sorted according to pseudotime beforehand (Sanchez-Castillo et al., 2017). Then it models the fold changes in gene expression between successive time points as a linear combination from the expression of gene regulators at the immediate previous time sample within the Bayesian network. The reconstruction of GRNs for early embryogenesis in mice and kidney cells of Danio rerio using this method allowed for the identification of hubs and the formation of hypotheses about differentiation regulators.

The HBFM method is based on gene co-expression analysis that employs a sparse hierarchical Bayesian factor model to reduce the impact of high intercellular variability and noise in single-cell datasets on the predicted network (Sekula et al., 2020). When analyzing single-cell transcriptomes from mouse brains, the program identified a significant number of known and putative protein-protein interactions from the STRING database.

Logical networks

While the previously presented methods infer networks that describe the regulatory effects of individual TFs, they do not account for the logical rules governing the combinatorial effect of multiple TFs on the expression of a target gene (Nguyen et al., 2021). For example, regulatory mechanisms may involve the activation of a gene only in the presence of several specific TFs or, alternatively, its inhibition by another TF regardless of additional factors. Boolean networks are capable of characterizing these combinations of interactions by representing the active or inactive state of a gene as a binary variable, discretized using a gene expression threshold, and combining these states using AND, OR, and NOT operations to explain the expression of all genes in the system.

The SCNS program computes logical rules that explain the progression of gene expression from one pseudotime point to another (Woodhouse et al., 2018). Application of this program to transcriptomes from early-stage human embryo cells resulted in reconstruction of a core GRN for preimplantation embryonic development. The LogicNet algorithm employs probabilistic continuous logic to build a Boolean network, in which gene expression is modeled as a continuous rather than binary variable between 0 and 1, allowing for the construction of GRNs with directed and signed edges (Malekpour et al., 2020). Using LogicNet, GRNs for early embryogenesis in mice were constructed.

Dif﻿ferential equations

The presence of pseudotime information in scRNA-seq data allows for modeling gene expression using ordinary differential equations (ODEs) (Nguyen et al., 2021). Here, the rate of expression changes for a target gene is a function of expression of the gene encoding its TF regulator. By solving this system of equations, regulatory relationships can be determined based on the weight of each TF in the function, which describes changes in gene expression. The SCODE algorithm makes a simplifying assumption that changes in gene expression can be defined as a linear combination of reduced dimensional spaces to effectively solve a less complex system of equations using linear regression (Matsumoto et al., 2017). Alternatively, GRISLI estimates the rate at which the expression of each gene changes according to the dynamic process in each cell (Aubin-Frankowski, Vert, 2020). It subsequently simplifies the system of equations based on the assumption that the inferred GRN has few regulatory edges compared to the number of genes in the network, thereby reducing the problem to sparse regression.

A valuable feature of GRISLI is that it allows cells to follow multiple differentiation trajectories, whereas most methods permit only a linear, non-branching trajectory. The DynGENIE3 algorithm applies the random forest approach of GENIE3 to solve a system of ODEs, where the change in the expression of one gene is defined as a potentially nonlinear combination of the expression of other genes (Huynh-Thu, Geurts, 2018).

Another class of approaches is based on the observation that variations in gene expression from cell to cell may arise from the stochastic nature of molecular regulatory interactions (Nguyen et al., 2021). The piecewise-deterministic Markov process (PDMP) defines ODEs for gene expression as a function of a stochastic two-state Markov process indicating whether the transcription of the gene is activated, rather than directly as a function of the expression of regulating TFs (Herbach et al., 2017).

For each gene, the probability function representing transitions between active and inactive states includes a weight for each potential regulator. PDMP uses maximum likelihood estimation to determine these weights and thus infers the edges of the GRN. The WASABI algorithm implements an alternative maximum likelihood estimation based on the concept that observed increases or decreases in gene expression should precede transitions between active and inactive states in an earlier time window (Bonnaffoux et al., 2019). The application of WASABI for reconstructing the GRN of erythrocyte differentiation in birds revealed its unusual properties of this GRN – absence of hubs, a distributed network structure, and control of the expression of most genes directly by the factor inducing differentiation.

## Refinement of GRNs reconstructed from scRNA-seq data through the recognition of TF binding sites

Despite the widespread use of scRNA-seq data for inferring GRNs, the accuracy of reconstructing the actual regulatory mechanisms based on these data remains unsatisfactory (Chen, Mar, 2018; Pratapa et al., 2020). This issue arises because programs for GRN inference from transcriptomic data are based on the assumption that the identified associations between the expression levels of TF-coding genes and their potential target genes imply direct transcriptional regulation. However, the observed associations may be caused by other biological phenomena or even random factors. Transcriptomic data do not contain direct information about regulatory events (e. g., TF binding to gene regulatory regions). Thus, it is challenging to distinguish between direct and indirect regulation based solely on scRNA-seq data.

To address these issues and enhance the effectiveness of GRN inference, it is necessary to incorporate additional data that directly characterize the factors involved in transcriptional regulation. For example, genome sequences bearing regulatory codes can be used to identify potential TF binding sites. In this case, the presence of a TF binding motif in the regulatory region of the target gene testifies in favor of direct TF-target gene regulation.

Accordingly, SCENIC utilizes a database of TF binding motifs to refine GRNs inferred with GENIE3 (Aibar et al., 2017). It keeps the links in the network only if the motifs, which correspond to the TF binding sites, are enriched in the promoter regions of the target genes. A later version, pySCENIC, employs parallelization to improve SCENIC efficiency (Van de Sande et al., 2020). In both studies, SCENIC successfully identified cell types in mouse and human brains (including those represented by as few as two to six cells), as well as stages of tumor development that are more difficult to distinguish than cell types (Aibar et al., 2017; Van de Sande et al., 2020). It also found a specific set of TFs for each cell type and tumor stage, including previously unknown oncological markers. The role of some of these markers in tumor progression was experimentally validated in the same studies.

## Integration of scRNA-seq and scATAC-seq data for GRN reconstruction

In the genome, DNA is packaged into nucleosomes – the basic structural units of chromatin, which hinder TF bind ing to DNA, thereby preventing gene transcription (Parmar, Padinhateeri, 2020). Activation of genes is only possible when their regulatory regions are free from nucleosomes. The nucleosomal packaging of DNA is a regulated process and varies depending on conditions and cell types. The scATAC- seq (single-cell Assay for Transposase-Accessible Chromatin using sequencing) technology allows for identification of open chromatin areas, i. e., DNA regulatory regions that are accessible for TF binding, in individual cells (Buenrostro et al., 2015). Thus, scATAC-seq data can contribute to a more accurate reconstruction of direct regulatory relationships between TFs and their targets in GRNs.

It has been shown that integrating bulk RNA-seq and ATAC-seq (or other epigenomic data) significantly enhances the accuracy of GRN inference (Qin et al., 2014; Wang et al., 2015; Ackermann et al., 2016). This methodology is also applicable to single-cell sequencing data. However, due to the specificity of transcriptomic and epigenomic profiles by cell type and conditions, combining RNA-seq data with ATAC-seq or ChIP-seq data typically requires that both datasets be obtained from cells of the same type under identical conditions.

Current technologies allow for simultaneous sequencing of the transcriptome and epigenome in the same cell (Angermueller et al., 2016; Hu et al., 2016; Chen et al., 2019). An alternative is the integration of scRNA-seq and scATAC-seq data obtained from different biological samples of the same nature. In this case, an additional challenge for GRN reconstruction is establishing the correspondence between cell clusters representing the same type, condition, or state across two types of sequencing data. So-called diagonal integration methods are being developed to address this challenge (Argelaguet et al., 2021).

Since scATAC-seq is most frequently used for epigenome profiling in individual cells, several bioinformatics tools have been developed to integrate scRNA-seq and scATAC-seq data for GRN inference (Loers, Vermeirssen, 2024). GRNs reconstructed based on these data are specifically referred to as enhancer GRNs (eGRNs). STREAM reconstructs eGRNs based on jointly profiled scRNA-seq and scATAC-seq data, using a Steiner tree problem model, a hybrid biclustering pipeline, and submodular optimization to infer gene networks (Li et al., 2024). STREAM has been tested on single-cell data from human organs with pathologies (Alzheimer’s disease and lymphocytic lymphoma) and has demonstrated its effectiveness in reconstructing TF–open binding site–gene connections along a pseudotime trajectory and in identifying transcriptional regulations specific to these diseases.

There are also programs that utilize the results of preliminary separate analyses of scRNA-seq and scATAC-seq data. For example, scMTNI takes as input a cell differentiation scheme, scRNA-seq results, and prior networks based on scATAC-seq for each cell type (Zhang et al., 2023). The application of scMTNI to scRNA-seq and scATAC-seq data on cell reprogramming in mice and differentiation of human hematopoietic cells allowed for the construction of eGRNs for both linear and branching lineages and the identification of regulators and other components of eGRNs specific to their fate transitions.

## Conclusion

The identification of gene relationships in regulation of their expression is a key to understanding the mechanisms that ensure the realization of genetic information into specific phenotypic traits. The reconstruction of GRNs based on omics data from individual cells provides a unique opportunity to systematically investigate the mechanisms of cellular differentiation, as it theoretically allows for the reconstruction of regulatory gene networks for specific cell types and even at distinct stages of their development. To date, a number of methods have been worked out for reconstructing such GRNs, many of which are available to users as a software. However, despite the promising nature of this approach, its potential has not yet been fully realized. Not all available methods are user-friendly or easy to interpret.

The shortage of methods for verifying the reconstructed GRNs is also an ongoing challenge. Perhaps for this reason, the use of these models in specific biological studies remains limited, and there are only a handful of successful applications of single cell GRNs to address biological questions. Further advancements in molecular genetic technologies for studying individual cells and computational methods for analyzing the data they generate (particularly for the purpose of reconstructing and analyzing GRNs) will significantly narrow the gap between our knowledge of the molecular determinants of traits (including at the cellular level) and the transcriptional cascades triggered by external or internal stimuli. Breakthrough discoveries made with GRNs reconstructed from single cell omics data are likely awaiting us in the future.

## Conflict of interest

The authors declare no conflict of interest.
